# Immune-Related Adverse Events After Immune Checkpoint Inhibitors for Melanoma Among Older Adults

**DOI:** 10.1001/jamanetworkopen.2022.3461

**Published:** 2022-03-22

**Authors:** Sara J. Schonfeld, Margaret A. Tucker, Eric A. Engels, Graça M. Dores, Joshua N. Sampson, Meredith S. Shiels, Stephen J. Chanock, Lindsay M. Morton

**Affiliations:** 1 Division of Cancer Epidemiology and Genetics, National Cancer Institute, National Institutes of Health, Department of Health and Human Services, Bethesda, Maryland; 2 Office of Surveillance and Epidemiology, Center for Drug Evaluation and Research, US Food and Drug Administration, Silver Spring, Maryland

## Abstract

**Importance:**

Immune checkpoint inhibitors (ICIs) have improved survival in patients with advanced melanoma but can be associated with a spectrum of immune-related adverse events (AEs), including both autoimmune-related AEs and other immune-related inflammatory AEs. These associations have primarily been evaluated in clinical trials that include highly selected patients, with older adults often underrepresented.

**Objective:**

To evaluate the association between use of ICIs and immune-related AEs (autoimmune and other immune related) among older patients with cutaneous melanoma.

**Design, Setting, and Participants:**

A population-based cohort study was conducted from January 1, 2011, to December 31, 2015. Data were analyzed from January 31 to May 31, 2021. With use of a linked database of Medicare claims and Surveillance, Epidemiology, and End Results (SEER) Program population-based cancer registries, patients of White race diagnosed with stages II-IV or unknown (American Joint Committee on Cancer, *AJCC Cancer Staging Manual* 6th edition) first primary cutaneous melanoma during 2011-2015, as reported to SEER, and followed up through December 31, 2015, were identified.

**Exposures:**

Immune checkpoint inhibitors for treatment of melanoma.

**Main Outcomes and Measures:**

The association between ICIs and immune-related AEs ascertained from Medicare claims data was estimated using multivariable Cox regression with hazard ratios (HRs) and 95% CIs and with cumulative incidence accounting for competing risk of death.

**Results:**

The study included 4489 patients of White race with first primary melanoma (3002 men [66.9%]; median age, 74.9 [range, 66.0-84.9] years). During follow-up (median, 1.4 [range, 0-5.0] years), 1576 patients (35.1%) had an immune-related AE on a Medicare claim. Use of ICIs (reported for 418 patients) was associated with autoimmune-related AEs (HR, 2.5; 95% CI, 1.6-4.0), including primary adrenal insufficiency (HR, 9.9; 95% CI, 4.5-21.5) and ulcerative colitis (HR, 8.6; 95% CI, 2.8-26.3). Immune checkpoint inhibitors also were associated with other immune-related AEs (HR, 2.2; 95% CI, 1.7-2.8), including Cushing syndrome (HR, 11.8; 95% CI, 1.4-97.2), hyperthyroidism (HR, 6.3; 95% CI, 2.0-19.5), hypothyroidism (HR, 3.8; 95% CI, 2.4-6.1), hypopituitarism (HR, 19.8; 95% CI, 5.4-72.9), other pituitary gland disorders (HR, 6.0; 95% CI, 1.2-30.2), diarrhea (HR, 3.5; 95% CI, 2.5-4.9), and sepsis or septicemia (HR, 2.2; 95% CI, 1.4-3.3). Most associations were pronounced within 6 months following the first ICI claim and comparable with or without a baseline history of autoimmune disease. The cumulative incidence at 6 months following the first ICI claim was 13.7% (95% CI, 9.7%-18.3%) for autoimmune-related AEs and 46.8% (95% CI, 40.7%-52.7%) for other immune-related AEs.

**Conclusions and Relevance:**

In this cohort study of older adults with melanoma, ICIs were associated with autoimmune-related AEs and other immune-related AEs. Although some findings were consistent with clinical trials of ICIs, others warrant further investigation. As ICI use continues to expand rapidly, ongoing investigation of the spectrum of immune-related AEs may optimize management of disease in patients.

## Key Points

**Question:**

What is the association between immune checkpoint inhibitors (ICIs) and immune-related adverse events (AEs), including both autoimmune and other immune-related AEs, among older patients with melanoma?

**Findings:**

In this population-based cohort study of 4489 older adults with stages II-IV or unknown cutaneous melanoma, the 418 patients who received ICIs had greater risk of developing autoimmune-related and other immune-related AEs, primarily affecting the endocrine and gastrointestinal systems compared with those who did not receive ICIs. The cumulative incidence at 6 months following first receipt of ICIs was 13.7% for autoimmune-related and 46.8% for other immune-related AEs.

**Meaning:**

This study found an association between treatment with ICIs and immune-related AEs among older adults with melanoma; further study is needed to determine the frequency of such AEs in this population.

## Teaser

This cohort study examines the association between use of immune checkpoint inhibitors and development of immune-related adverse events in older adults with melanoma.

## Introduction

Immune checkpoint inhibitors (ICIs) have substantially altered treatment of advanced melanoma in the past decade.^1^ However, clinical trials have reported a wide spectrum of immune-related adverse events (AEs) among patients with melanoma treated with ICIs, most commonly dermatologic, endocrine, gastrointestinal, and hepatic but also rarer neurologic, urologic, pulmonary, and cardiac outcomes.^2,3,4,5,6,7,8,9,10^ This spectrum includes both autoimmune diseases (autoimmune-related AEs) and inflammatory conditions not uniquely attributed to an autoimmune cause (other immune-related AEs).^5^ Because ICI use has been incorporated into standard community practice, improved understanding of immune-related AEs in general population-based settings is needed, particularly among older adults. Melanoma incidence increases with age,^11^ yet older adults are often underrepresented in clinical trials.^12^ We evaluated the association between ICIs and autoimmune- and other immune-related AEs within a cohort of older patients with melanoma using the US Surveillance, Epidemiology, and End Results (SEER)–Medicare linked database.^13^

## Methods

The population-based cohort study included patients of White race with Medicare coverage (US health care system for adults aged ≥65 years) who were diagnosed with incident first primary cutaneous melanoma (American Joint Committee on Cancer, *AJCC Cancer Staging Manual* 6th edition, excluding stage I) at ages 66 to 84 years during January 1, 2011, to December 31, 2015, as reported to 17 SEER registries (eFigure 1 in the Supplement). This research was waived from ethics committee review by the National Institutes of Health Office of Human Subjects Research because it relied on deidentified existing data. This study followed the Strengthening the Reporting of Observational Studies in Epidemiology (STROBE) reporting guideline for cohort studies.

We restricted our analysis to individuals younger than 85 years at diagnosis of the first primary melanoma because of concerns about potential underascertainment of cancer and other medical conditions among the older population.^14^ We excluded patients with stage I melanoma because less than 1% received ICIs and excluded patients not of White race owing to small numbers. To optimize ascertainment of treatment and medical conditions from claims data, we required 12 months or more of Medicare Part A (inpatient hospital, skilled nursing facility, and hospice care), Medicare Part B (physician and outpatient services), and non–health maintenance organization Medicare coverage before and after melanoma diagnosis (or continuous coverage until death for individuals surviving <12 months).

Immune checkpoint inhibitors, other cancer treatments, and immune-related AEs were ascertained from inpatient and outpatient Medicare claims using *International Classification of Diseases, Ninth Revision* and Healthcare Common Procedure Coding System codes (eTable 1 and eTable 2 in the Supplement). For each immune-related AE, patients were followed up from melanoma diagnosis until the earliest of the first claim after melanoma diagnosis for that immune-related AE or the end of the follow-up period (second cancer diagnosis in SEER, age 85 years, death, date of last claims available, or analysis end [December 31, 2015]). We categorized immune-related AEs according to conditions of autoimmune cause (autoimmune-related AEs) and conditions not uniquely attributed to an autoimmune cause (other immune-related AEs). We further classified immune-related AEs as transient or chronic (eTable 2 in the Supplement) and restricted analyses of chronic diseases to individuals without a known baseline history of that disease.

### Statistical Analysis

Analysis was performed using multivariable Cox proportional hazards regression (person-year time scale) with estimated hazard ratios (HRs) and 95% CIs for the association between ICIs (time-dependent) and each immune-related AE (SAS, version 9.4; SAS Institute Inc). All models were stratified by calendar year of melanoma diagnosis and adjusted for baseline categorical variables (age at melanoma diagnosis, sex, stage at melanoma diagnosis, and National Cancer Institute comorbidity index) and time-dependent categorical variables for history of autoimmune and nonautoimmune disease (as defined in eTable 2 in the Supplement), chemotherapy, radiotherapy, and other types of immunotherapy. Models of transient outcomes were further adjusted for baseline history of that disease.

We also estimated the cumulative incidence of immune-related AEs accounting for competing risk of death by ICI receipt (Stata, version 15.1; StataCorp LLC).^15^ In these analyses, the cumulative incidence after ICI included follow-up from the date of the first ICI claim until the earliest of the event of interest or end of follow-up. Cumulative incidence in the absence of an ICI claim (for patients who never had a claim for an ICI) or before the first ICI claim included follow-up from the date of melanoma diagnosis until the earliest of the first ICI claim, the event of interest, or end of follow-up. Statistical significance for all analyses was considered a 2-sided *P* < .05. Data analysis was conducted from January 31 to May 31, 2021.

## Results

Among 4489 patients of White race with first primary melanoma (1487 women [33.1%]; 3002 men [66.9%]; median age, 74.9 [range, 66.0-84.9] years), 418 individuals (9.3%) had an ICI Medicare claim during follow-up. Among this group, 314 patients (75.1%) received only ipilimumab (Table 1). Use of ICIs was more common among individuals with advanced-stage melanoma and those who also received other treatments. The median time from melanoma diagnosis to first ICI claim was 0.7 years (maximum, 4.0 years; IQR, 0.3-1.4 years).

**Table 1.** Characteristics of Patients Diagnosed With First Primary Melanoma During 2011-2015, Overall and by Receipt of ICIs^a^

Characteristic	Receipt of ICI during follow-up, No. (%)^b^		
	Total (N = 4489)	None (n = 4071)	Any (n = 418)^c^
Calendar year of melanoma			
2011	859 (19.1)	781 (90.9)	78 (9.1)
2012	843 (18.8)	757 (89.8)	86 (10.2)
2013	883 (19.7)	779 (88.2)	104 (11.8)
2014	937 (20.9)	836 (89.2)	101 (10.8)
2015	967 (21.5)	918 (94.9)	49 (5.1)
Stage at melanoma diagnosis			
II	1832 (40.8)	1748 (95.4)	84 (4.6)
III	810 (18)	659 (81.4)	151 (18.6)
IV	529 (11.8)	376 (71.1)	153 (28.9)
Unknown	1318 (29.4)	1288 (97.7)	30 (2.3)
Age at melanoma, y			
66-69	1035 (23.1)	931 (90)	104 (10)
70-74	1239 (27.6)	1096 (88.5)	143 (11.5)
75-79	1147 (25.6)	1052 (91.7)	95 (8.3)
80-84	1068 (23.8)	992 (92.9)	76 (7.1)
Sex			
Male	3002 (66.9)	2711 (90.3)	291 (9.7)
Female	1487 (33.1)	1360 (91.5)	127 (8.5)
NCI Comorbidity Index^d^			
0	2413 (53.8)	2166 (89.8)	247 (10.2)
>0 to <1.69	901 (20.1)	823 (91.3)	78 (8.7)
≥1.69	1175 (26.2)	1082 (92.1)	93 (7.9)
Previous immune-related disease^e^			
Autoimmune disease			
No/unknown	3114 (69.4)	2827 (89.8)	287 (9.2)
Yes	1375 (30.6)	1244 (90.5)	131 (9.5)
Other immune-related disease			
No/unknown	2128 (47.4)	1911 (89.8)	217 (10.2)
Yes	2361 (52.6)	2160 (91.5)	201 (8.5)
Receipt of other cancer therapy during study follow-up^f^			
Radiotherapy			
No/unknown	3874 (86.3)	3629 (93.7)	245 (6.3)
Yes	615 (13.7)	442 (71.9)	173 (28.1)
Chemotherapy			
No/unknown	3916 (87.2)	3821 (97.6)	95 (2.4)
Yes	573 (12.8)	250 (43.6)	323 (56.4)
Cytokine immunotherapy			
No/unknown	4419 (98.4)	4021 (91)	398 (9)
Yes	70 (1.6)	50 (71.4)	20 (28.6)
Other/unspecified immunotherapy			
No/unknown	4287 (95.5)	4021 (93.8)	266 (6.2)
Yes	202 (4.5)	50 (24.8)	152 (75.2)

Abbreviation: ICI, immune checkpoint inhibitor.

^a^ Study population restricted to patients of White race with cutaneous melanoma at stages II-IV or unknown (American Joint Committee on Cancer, *AJCC Cancer Staging Manual* 6th edition).

^b^ Patients were followed up until the earliest diagnosis of the second malignant neoplasm, age 85 years, death, date of last claims available, or end of analysis (December 31, 2015).

^c^ Type of ICI received during follow-up: ipilimumab only (n = 314); ipilimumab with nivolumab (n = 11); ipilimumab with pembrolizumab (n = 45); pembrolizumab (n = 36); ipilimumab, nivolumab, and pembrolizumab (n <11); nivolumab (n <11), and nivolumab and pembrolizumab (n <11).

^d^ National Cancer Institute (NCI) comorbidity index calculated from Medicare claims in the year before melanoma diagnosis. Cut points were based on the median value among people with non-0 values in the full study population.

^e^ Based on claims at or before date of melanoma diagnosis; eTable 2 in the Supplement provides a list of autoimmune and other diseases.

^f^ Includes treatments received before, with, or after use of ICIs. Treatments, including ICI, were classified as yes if first claim was between 90 days before diagnosis of melanoma and up to (inclusive of) exit. Claims were searched beginning 90 days before melanoma diagnosis (treatment received before the diagnosis date in Surveillance, Epidemiology, and End Results Program cancer registries of the first primary melanoma reflects delays between clinical diagnosis and pathological report). eTable 1 in the Supplement provides specific treatment codes.

During follow-up (median, 1.4 [range, 0-5.0] years), 35.1% (1576 of 4489) of patients had at least 1 immune-related AE based on Medicare claims. Among 49.5% of patients (207 of 418) who developed an immune-related AE after ICI receipt, 25.1% (52 of 207) had 2 different immune-related AEs and 22.7% (47 of 207) had more than 2 immune-related AEs. The most common post-ICI immune-related AEs were diarrhea (n = 95), sepsis or septicemia (n = 59), hypothyroidism (n = 54), and primary adrenal insufficiency (n = 34).

Use of ICIs was associated with statistically significant increased risks for autoimmune-related AEs (HR, 2.5; 95% CI, 1.6-4.0), including primary adrenal insufficiency (HR, 9.9; 95% CI, 4.5-21.5) and ulcerative colitis (HR, 8.6; 95% CI, 2.8-26.3) (Table 2). Use of ICIs also was associated with other immune-related AEs (HR, 2.2; 95% CI, 1.7-2.8), including Cushing syndrome (HR, 11.8; 95% CI, 1.4-97.2), hyperthyroidism (HR, 6.3; 95% CI, 2.0-19.5), hypothyroidism (HR, 3.8; 95% CI, 2.4-6.1), hypopituitarism (HR, 19.8; 95% CI, 5.4-72.9), other pituitary gland disorders (HR, 6.0; 95% CI, 1.2-30.2), diarrhea (HR, 3.5; 95% CI, 2.5-4.9), and sepsis or septicemia (HR, 2.2; 95% CI, 1.4-3.3). The remaining autoimmune- and other immune-related AEs investigated were not associated with ICIs, generally based on small event numbers. In sensitivity analyses, results were similar when we extended the duration of required Medicare coverage from 12 months to 18, 24, or 30 months before and after melanoma diagnosis; when we removed adjustment for comorbidities and broad categories of autoimmune and nonautoimmune disease from the Cox proportional hazards regression models; when we further adjusted models for Breslow depth; and when we removed age 85 years as a censoring criterion.

**Table 2.** Immune-Related AEs After ICIs Among Patients Diagnosed With First Primary Melanoma During 2011-2015^a^

Immune-related AEs^b^	No. included in analysis^c^	No ICI or event prior to ICI [reference], No. of events^d^	After ICI		
			No. of events^d^	HR (95% CI)^e^	Wald *P* value
Autoimmune-related AEs	3114	304	45	2.5 (1.6-4.0)	<.001
Endocrine	4420	50	38	8.8 (4.3-18.0)	<.001
Primary adrenal insufficiency	4460	39	34	9.9 (4.5-21.5)	<.001
Gastrointestinal	4219	73	20	3.5 (1.6-7.6)	.001
Regional enteritis/Crohn disease	4435	<11	<11	3.9 (0.9-17.1)	.07
Ulcerative colitis	4417	26	15	8.6 (2.8-26.3)	<.001
Miscellaneous					
Asthma	3858	142	<11	0.7 (0.2-1.9)	.46
Other immune-related AEs	4489	1712	146	2.2 (1.7-2.8)	<.001
Endocrine	3022	271	52	3.3 (2.0-5.2)	<.001
Cushing syndrome	4477	<11	<11	11.8 (1.4-97.2)	.02
Thyrotoxicosis with or without goiter (hyperthyroidism)	4283	40	<11	6.3 (2.0-19.5)	.001
Hypopituitarism	4479	11	14	19.8 (5.4-72.9)	<.001
Hypothyroidism	3093	262	54	3.8 (2.4-6.1)	<.001
Other disorders of pituitary gland (includes hypophysitis)	4483	<11	<11	6.0 (1.2-30.2)	.03
Gastrointestinal	4489	501	106	3.0 (2.2-4.1)	<.001
Gastroenteritis and colitis, excluding ulcerative colitis	4489	31	<11	2.2 (0.7-6.7)	.17
Diarrhea	4489	404	95	3.5 (2.5-4.9)	<.001
Stomatitis and mucositis (including ulcerative, aphthous)	4489	37	<11	1.3 (0.4-3.8)	.66
Myalgia and myositis, not otherwise specified	4489	311	17	1.5 (0.8-2.9)	.20
Vitiligo	4477	14	<11	2.1 (0.5-8.3)	.30
Septicemia, sepsis	4489	280	59	2.2 (1.4-3.3)	<.001

Abbreviations: AEs, adverse events; HR, hazard ratio; ICI, immune checkpoint inhibitor.

^a^ Study population restricted to patients of White race diagnosed with American Joint Committee on Cancer stages II-IV or unknown stage cutaneous melanoma during 2011-2015 as identified in the Surveillance, Epidemiology, and End Results-Medicare linked database.

^b^ Outcomes for which there were at least 5 events in each group are presented. The full list of immune-related AEs is found in eTable 2 in the Supplement; HRs are not presented because counts were too small for models to be stable.

^c^ Immune-related AEs were designated as chronic or transient. For chronic diseases, it is assumed that an individual can have only 1 incident diagnosis per lifetime. For transient diseases, it is assumed that an individual can have multiple incident events per lifetime. Variation is seen in the analytic sample size for chronic diseases (designated in eTable 2 in the Supplement) owing to exclusion of individuals with a baseline history of the disease. For models of transient diseases, individuals with a baseline history of the diseases were included in analyses; therefore, the total sample size remains fixed.

^d^ Number of events captures the number of people with a claim for the event following melanoma diagnosis. The exact number is not shown if there were less than 11 events to protect patient confidentiality.

^e^ Hazard ratios and 95% CIs estimated from multivariable Cox proportional hazards regression with person-years as the time scale and stratified by calendar year of melanoma diagnosis. All models were adjusted for age at melanoma diagnosis (66-69, 70-74, 75-79, or 80-84), sex, stage at melanoma diagnosis, and NCI comorbidity index (0, >0-1 -<1.69, ≥1.69; cut points were derived from individuals with non-0 values), and time-dependent variables for history of autoimmune and nonautoimmune disease and for chemotherapy, radiotherapy, and other types of immunotherapy. Models of transient outcomes were further adjusted for baseline history of that disease.

Additional analyses for immune-related AEs with 11 or more events following receipt of ICIs showed that most HRs were statistically significantly higher less than 6 vs greater than or equal to 6 months following first receipt of an ICI (eTable 3 in the Supplement). For most immune-related AE types, outcomes first appeared on claims within 3 months of first ICI receipt; the exceptions were primary adrenal insufficiency, hypopituitarism, and myalgia/myositis not otherwise specified. In sensitivity analyses, results were similar when restricted to patients treated with ipilimumab only or those with stages III-IV melanoma. Hazard ratios were generally comparable for individuals with and without a baseline history of autoimmune disease (eTable 3 in the Supplement).

Six months following the first ICI claim, the cumulative incidence for autoimmune-related AEs was 13.7% (95% CI, 9.7%-18.3%) and for other immune-related AEs, 46.8% (95% CI, 40.7-52.7), increasing to 18.5% (95% CI, 13.7%-23.8%) for autoimmune-related AEs and 52.0% (95% CI, 45.7%-57.9%) for other immune-related AEs at 1 year (Figure; eTable 4 in the Supplement). Substantially lower estimates were observed in the absence of or before ICI receipt (6 months: autoimmune-related AEs, 4.5%; 95% CI, 3.8%-5.3%; other immune-related AEs, 24.3%; 95% CI, 23.0%-25.6%). Cumulative incidence estimates were similar after restricting analysis to patients with stages III-IV melanoma (eFigure 2 and eTable 4 in the Supplement).

**Figure.**
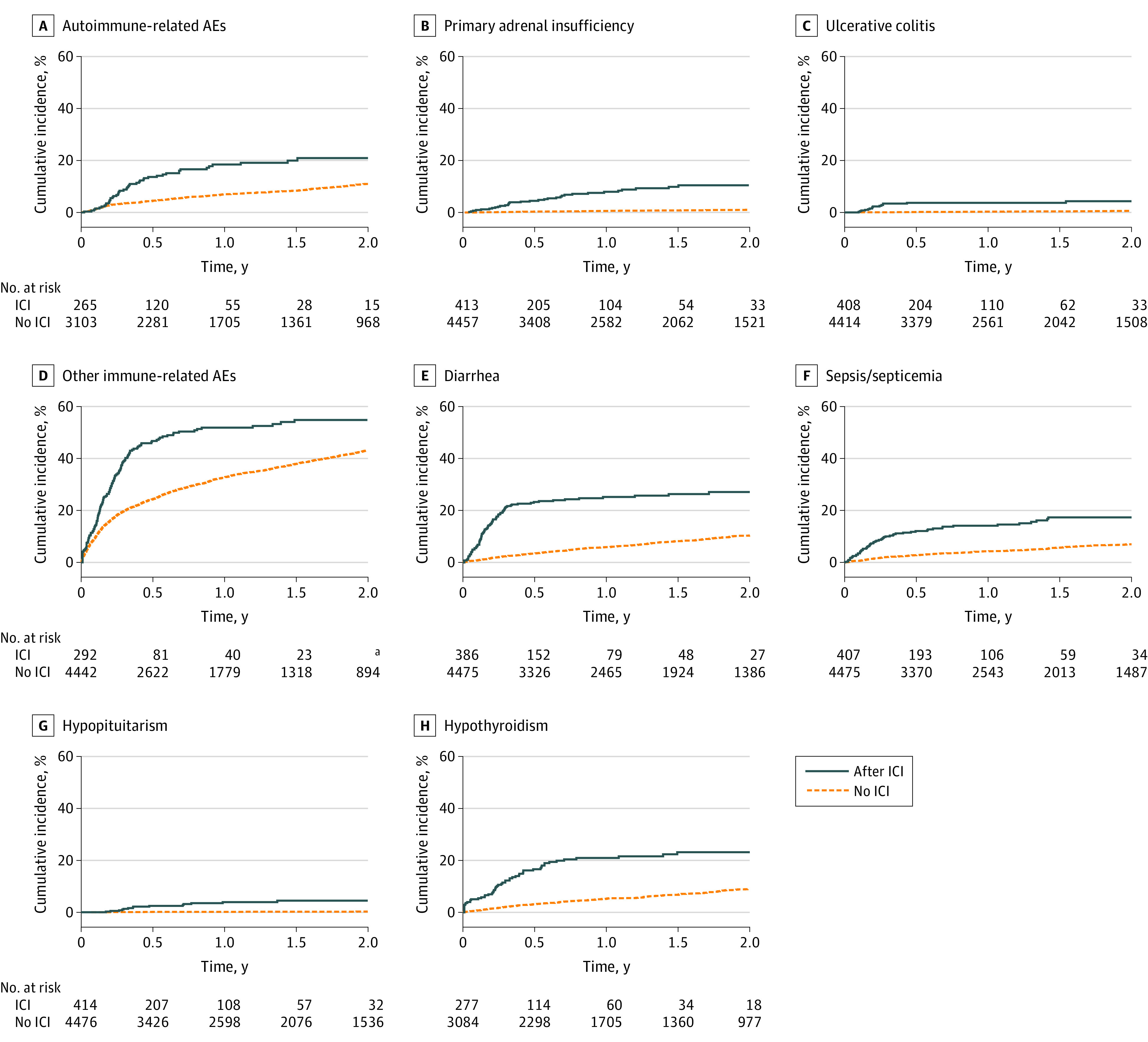
Cumulative Incidence of Selected Autoimmune- and Other Immune-Related Adverse Events (AEs) Among Patients Diagnosed With Cutaneous Melanoma, Accounting for Competing Risk of Death, by Receipt of Immune Checkpoint Inhibitors (ICIs) eTable 2 in the Supplement provides the definition of autoimmune-related AEs (A-C) and other immune-related AEs (D-H). Cumulative incidence in the absence of ICIs is provided for perspective, but the curves have different time scales and are not adjusted for differences between the populations and should therefore be interpreted cautiously. eTable 4 in the Supplement provides point estimates and 95% confidence bounds. Individuals with a previous claim (at or before melanoma diagnosis) were excluded from analyses of autoimmune-related AEs (overall), primary adrenal insufficiency, ulcerative colitis, hypothyroidism, and hypopituitarism as described in eTable 2 in the Supplement. For all outcomes, the number at risk at the start of an interval excludes people who had the event earlier in follow-up, died, or ended follow-up before the start of the interval. ^a^Small numbers were suppressed for privacy.

## Discussion

We used large-scale medical claims data linked with cancer registry data to provide a population-based perspective of the association between use of ICIs and immune-related AEs among older patients of White race with melanoma. The use of ICIs was associated with increased risks of autoimmune-related and other immune-related AEs, with the highest autoimmune-related risks due to primary adrenal insufficiency and ulcerative colitis. For other-immune-related AEs, most of the investigated endocrinopathies, diarrhea, and sepsis or septicemia were all associated with ICI receipt. Although based on small numbers, individuals with a known baseline history of autoimmune disease did not clearly have higher immune-related AE risks than those without. Generally, HRs were higher less than 6 months after ICI receipt and no longer significantly elevated thereafter, although increased risks of primary adrenal insufficiency and hypopituitarism persisted. The 6-month cumulative incidence estimate following ICI receipt for autoimmune-related AEs was 13.7% and for other immune-related AEs, 46.8%,

The magnitude of the primary adrenal insufficiency and ulcerative colitis risks were higher than expected^2,3,16,17,18,19^ and warrant study in settings with more detailed medical history and clinical diagnostic data. Although primary adrenal insufficiency is an established immune-related AE following administration of ICIs, secondary adrenal insufficiency due to hypophysitis has been more commonly reported.^2,3,16,17^ Similarly, nonulcerative colitis has been more commonly reported than ulcerative colitis.^2,3,17,18,19^ Our findings could reflect outcome misclassification owing to confusion between primary and secondary adrenal insufficiency and between ulcerative colitis and other forms of colitis. Because baseline medical histories were likely incomplete (no information before age 65 years), it is also possible that some of the increased risk for ulcerative colitis reflected a flare of preexisting disease.

Overall, other immune-related AEs were more common than autoimmune-related AEs as demonstrated by the cumulative incidence curves. The risks observed in this older population between ICI use and diarrhea, hypothyroidism, hyperthyroidism, and hypopituitarism are consistent with clinical trial reports for ipilimumab (the predominant ICI in our study).^2,3,16,17,18^ The observed increased risks for sepsis or septicemia and Cushing syndrome are less clear because these conditions have been reported as very rare events following use of ICIs.^2,3,20^ Potentially, immunosuppressive therapy administered for earlier immune-related AEs could explain the observed increased risk, but we did not capture that treatment information.

We did not confirm previously reported associations between use of ICIs and vitiligo, hepatitis, myocarditis, or pneumonitis,^5^ possibly owing to small sample size, which would particularly affect rarer outcomes (eg, pneumonitis and myocarditis) among patients with melanoma. Furthermore, our reliance on claims data—which only capture outcomes that require medical intervention and yield a diagnosis—may have led to underascertainment of vitiligo.

### Strengths and Limitations

Key strengths of our study include the general population setting consisting of older individuals, which is a population often underrepresented in clinical trials.^12^ The median age was 75 years at the start of follow-up in our study compared with median or mean ages ranging from 51 to 66 years in clinical trials^2,3,4,7,8,9,10,17^ In addition, we quantified both relative and absolute risks for a broad spectrum of autoimmune-related AEs and other immune-related AEs and, in contrast to most previous observational studies, included an internal comparison. Although sample size was limited, the exploratory analyses by baseline history of autoimmune disease contribute to an area of ongoing debate in the literature, in part because patients with a history of severe autoimmune disease were historically excluded from clinical trials of ICIs.^21^

The study has limitations. The limitations include small sample size to investigate differences by finer categories of time since first ICI, sex,^22^ or other factors (eg, age); potential for increased surveillance among patients receiving ICIs,^23^ which would have inflated our risk estimates; lack of diagnostic specificity in the *International Classification of Diseases, Ninth Revision*; and predominance of patients treated with ipilimumab alone, which contrasts with current practice.^1^ Limitations related to the use of claims data include the absence of information on services received outside of the US or those covered by other insurance plans^13^ and variability in the quality of claims data by reimbursement levels.^24^ The latter may favor the accurate ascertainment of ICIs and other expensive antineoplastic treatment but reduce ascertainment of health conditions associated with limited reimbursement. In addition, in the absence of medical records, we could not assess for consistency of diagnostic criteria across claims. These potential sources of misclassification would need to be differential by patient subgroup to bias our HRs, which seems unlikely.

As another limitation, our results may not be representative of all individuals older than 65 years diagnosed with melanoma (and provide no information about younger patients) because our population was predominantly male, restricted to White race, and excluded patients who were enrolled in managed care plans because they lack patient-level claims.^13^ Most patients who received ICIs also received other treatments. Although we adjusted for other treatments in our Cox proportional hazards regression models, the magnitude of HRs and cumulative incidence estimates reported herein may not be generalizable following treatment with ICIs alone.

## Conclusions

Although some associations we observed are consistent with clinical trial findings, others warrant further investigation to understand the factors that may be associated with the observed differences in results between these study populations. As ICI use continues to expand rapidly because of the effectiveness in treating advanced melanoma^1^ and other cancers,^23^ continued investigation of the spectrum of immune-related AEs in various at-risk populations is essential for optimizing management of disease in patients.

## Notes

Supplement. **eFigure 1.** Selection of Analytic Cohort **eTable 1.** Healthcare Common Procedure Coding System (HCPCS) and *International Classification of Diseases-9th Revision* (*ICD-9*) Codes for Identifying Immunotherapy, Chemotherapy and Radiotherapy From Medicare Claims **eTable 2.** *International Classification of Diseases-9th Revision* (*ICD-9*) Codes for Identifying Immune-Related Adverse Events (AEs) **eTable 3.** Hazard Ratios for Selected Immune-Related Adverse Events (AEs) After Ipilimumab Only and After Any Immune Checkpoint Inhibitor (ICI) - Among Patients Diagnosed With Stages III-IV Melanoma, by Time Since First ICI, and by Baseline History of Autoimmune Disease **eTable 4. **Cumulative Incidence of Selected Immune-Related Adverse Events (AEs) by Time (Years) Accounting for Competing Risk of Death **eFigure 2.** Cumulative Incidence of Selected Autoimmune- and Other Immune-Related Adverse Events (AEs) Among Patients Diagnosed With Stages III-IV Cutaneous Melanoma, Accounting for Competing Risk of Death, by Receipt of Immune Checkpoint Inhibitors (ICIs)
