# Supplementary material for: Immune-Related Adverse Events After Immune Checkpoint Inhibitors for Melanoma Among Older Adults
Source: JAMA Netw Open. 2022 Mar 22;5(3):e223461. doi: 10.1001/jamanetworkopen.2022.3461 (PMC8941351; doi:10.1001/jamanetworkopen.2022.3461)
Supplement: Supplement. — eFigure 1. Selection of Analytic Cohort eTable 1. Healthcare Common Procedure Coding System (HCPCS) and International Classification of Diseases-9th Revision (ICD-9) Codes for Identifying Immunotherapy, Chemotherapy and Radiotherapy From Medicare Claims eTable 2. International Classification of Diseases-9th Revision (ICD-9) Codes for Identifying Immune-Related Adverse Events (AEs) eTable 3. Hazard Ratios for Selected Immune-Related Adverse Events (AEs) After Ipilimumab Only and After Any Immune Checkpoint Inhibitor (ICI) - Among Patients Diagnosed With Stages III-IV Melanoma, by Time Since First ICI, and by Baseline History of Autoimmune Disease eTable 4. Cumulative Incidence of Selected Immune-Related Adverse Events (AEs) by Time (Years) Accounting for Competing Risk of Death eFigure 2. Cumulative Incidence of Selected Autoimmune- and Other Immune-Related Adverse Events (AEs) Among Patients Diagnosed With Stages III-IV Cutaneous Melanoma, Accounting for Competing Risk of Death, by Receipt of Immune Checkpoint Inhibitors (ICIs) [file jamanetwopen-e223461-s001.pdf]

Supplementary Online Content

Schonfeld SJ, Tucker MA, Engels EA, et al. Immune-related adverse events after immune checkpoint inhibitors for melanoma among older adults. *JAMA Netw Open*. 2022;5(3):e223461. doi:10.1001/jamanetworkopen.2022.3461

eFigure 1. Selection of Analytic Cohort

eTable 1. Healthcare Common Procedure Coding System (HCPCS) and *International Classification of Diseases-9th Revision* (*ICD-9*) Codes for Identifying Immunotherapy, Chemotherapy and Radiotherapy From Medicare Claims

eTable 2. *International Classification of Diseases-9th Revision* (*ICD-9*) Codes for Identifying Immune-Related Adverse Events (AEs)

eTable 3. Hazard Ratios for Selected Immune-Related Adverse Events (AEs) After Ipilimumab Only and After Any Immune Checkpoint Inhibitor (ICI) - Among Patients Diagnosed With Stages III-IV Melanoma, by Time Since First ICI, and by Baseline History of Autoimmune Disease

eTable 4. Cumulative Incidence of Selected Immune-Related Adverse Events (AEs) by Time (Years) Accounting for Competing Risk of Death

eFigure 2. Cumulative Incidence of Selected Autoimmune- and Other Immune-Related Adverse Events (AEs) Among Patients Diagnosed With Stages III-IV Cutaneous Melanoma, Accounting for Competing Risk of Death, by Receipt of Immune Checkpoint Inhibitors (ICIs)

This supplementary material has been provided by the authors to give readers additional information about their work.

© 2022 Schonfeld SJ et al. *JAMA Network Open.*

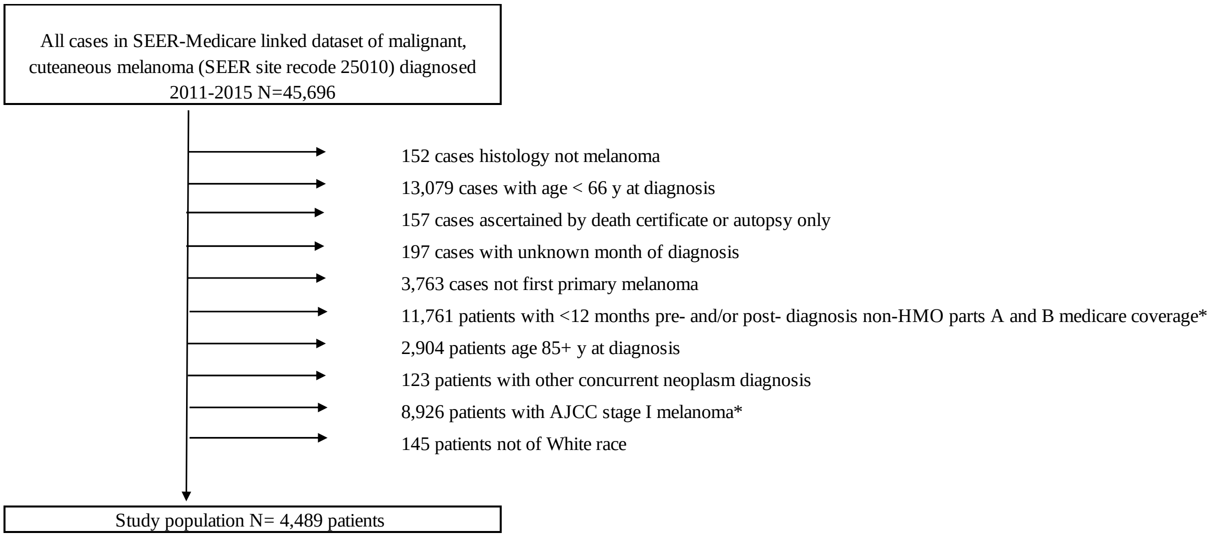

eFigure1. Selection of analytic cohort.
The starting year (2011) corresponds to the first immune checkpoint inhibitor approved for melanoma in the US. Numbers shown next to the arrows are the exclusions at each step. *Or coverage until death for people who survived < 12 months. Stage I excluded because <1% of these patients received an ICI.

© 2022 Schonfeld SJ et al. *JAMA Network Open.*

eTable 1. Healthcare Common Procedure Coding System (HCPCS) and International Classification of diseases-9th revision (ICD-9) codes for identifying immunotherapy, chemotherapy, and radiotherapy from Medicare claims[a]

**A. Immunotherapy**	**Codes[b]**
Checkpoint inhibitors	
Avelumab	C9491
Atezolizumab	C9483
Imlygic	C9472
Ipilimumab	C9284, J9228
Nivolumab	C9453, J9299
Pembrolizumab	C9027, J9271
Durvalumab	C9492
Cytokines	
Denileukin	C1084, J9160
IFN2A	J9213
IFN2B	J9214
IFNG2B	J9216
IFNAlphacon	J9212
IFNN3	J9215
IL2	00.15 (ICD-9 procedure), J9015
PegIFN2A	S0145
PegIFN2B	S0146, S0148
Other/unspecified	
BCG	C9416, J9031
TVEC	C9472, J9325
Unspecified	99.28 (ICD-9 procedure), S2107, V58.12 (ICD-9 diagnostic)
**B. Chemotherapy**	
Ado-trastuzumab emtansine	C9131, J9354
Aflibercept	J0178, J9400, Q2046
Alemtuzumab	C9110, J9010, S0087
Arsenic	C9012, J9017
Asparaginase	J9019, J9020
Azacitidine	J9025, C9218
Belinostat	J9032
Bendamustine	J9033
Bevacizumab	C9214, C9257, J9035, Q2024, S0116
Bleomycin	C9417, J9040
Blinatumomab	J9039
Bortezomib	C9207, J9041, S0115
Brentuximab	C9287, J9042

© 2022 Schonfeld SJ et al. *JAMA Network Open.*

Busulfan	C1178, J0594, J8510
Cabazitaxel	J9043
Capecitabine	J8520, J8521
Carboplatin	J9045
Carfilzomib	J9047
Carmustine	C9437, J9050
Cetuximab	C9215, J9055
Chlorambucil	S0172
Cisplatin	C9418, J9060, J9062
Cladribine	C9419, J9065
Clofarabine	J9027
Cyclophosphamide	C9420, C9421, J8530, J9070, J9080, J9090, J9091, J9092, J9093, J9094, J9095, J9096, J9097
Cytarabine	C1166, C9422, J9098, J9100, J9110
Dacarbazine	C9423, J9130, J9140
Dactinomycin	J9120
Daunorubicin	C9424, J9150, J9151
Decitabine	J0894
Docetaxel	J9170, J9171
Doxorubicin	C9415, J9000, J9001, J9002, Q2048, Q2049, Q2050
Epirubicin	C1167, J9178, J9180
Eribulin	J9179
Etoposide	C9414, C9425, J8560, J9181, J9182
Everolimus	J7527, J8561
Floxuridine	C9426, J9200
Fludarabine	C9262, J8562, J9185
Fluorouracil	J9190
Gefitinib	J8565
Gemcitabine	J9201
Gemtuzumab	C9004, J9300
Hydroxyurea	S0176
Idarubicin	C9429, J9211
Ifosfamide	C9427, J9208
Imatinib	S0088
Irinotecan	J9206
Ixabepilone	J9207
Leucovorin	J0640
Levoleucovorin	J0641
Lomustine	S0178
Mechlorethamine	J9230
Melphalan	J8600, J9245
Methotrexate	J8610, J9250, J9260
Mitomycin	C9432, J9280, J9290, J9291
Mitoxantrone	J9293

© 2022 Schonfeld SJ et al. *JAMA Network Open.*

Nelarabine	J9261
Obinutuzumab	J9301
Ofatumumab	J9302
Omacetaxine	J9262
Oxaliplatin	C9205, J9263
Paclitaxel	C9431, J9264, J9265, J9267, C9127
Panitumumab	C9235, J9303
Pegasparagase	J9266
Pemetrexed	C9213, J9305
Pentostatin	J9268
Pertuzumab	J9306
Plicamycin	J9270
Pralatrexate	J9307
Procarbazine	S0182
Ramucirumab	J9308
Rituximab	J9310
Romidepsin	C9265, J9315
Sipuleucel	Q2043
Streptozocin	J9320
Temozolomide	C1086, J8700, J9328
Temsirolimus	J9330
Teniposide	Q2017
Thiotepa	C9433, J9340
Topotecan	J8705, J9350, J9351
Trastuzumab	J9355
Valrubicin	J9357
Vinblastine	J9360
Vincristine	J9370, J9371, J9375, J9380
Vinorelbine	C9440, J9390
**C. Radiotherapy**	
ICD-9 codes	Diagnostic: V58.0, V67.1, Procedure: 92.21-92.29
HCPCS Codes	77261-77299, 77300-77399, 77401-77499, 77520, 77523, 76370, 76950, 77750-77799, C1325, C1348, C1350, C1700-C1712, C1715-C1720, C1728, C1790-C1806, C2616, C2632, C2633, C9714, C9715, G0174, G0178, G0179, G0261, G0256, G0273, G0274, G0338-G0340

a. Medicare claims files (MEDPAR, Carrier claims (NCH), OUTPT, and DME) searched beginning 90 days prior to diagnosis of first primary melanoma
b. HCPCS unless otherwise specified

© 2022 Schonfeld SJ et al. *JAMA Network Open.*

eTable 2. International Classification of diseases-9th revision (ICD-9) codes for identifying immune-related adverse events (AEs)[a]

	Designation: Chronic or transient[b]	ICD-9 Codes
I. Autoimmune-related AEs	Chronic	
Systemic/Connective tissue	Chronic	
Ankylosing spondylitis	Chronic	720.0
Dermatomyositis/polymyositis	Chronic	710.3, 710.4
Felty's syndrome	Chronic	714.1
Systemic lupus erythematosus	Chronic	710.0
Polymyalgia rheumatica	Chronic	725
Reactive arthritis	Chronic	99.3
Rheumatoid arthritis	Chronic	714.0, 714.2
Sarcoidosis	Chronic	135
Sjogren's syndrome	Chronic	710.2
Systemic sclerosis/scleroderma	Chronic	710.1
Cardiovascular	Chronic	
Giant cell arteritis	Chronic	446.5
Polyarteritis nodosa and allied conditions (systemic vasculitis)	Chronic	446, 447.6
Endocrine	Chronic	
Primary adrenal insufficiency	Chronic	255.4
Chronic thyroiditis/Hashimoto thyroiditis	Chronic	245.2
Primary biliary cirrhosis	Chronic	571.6
Autoimmune hepatitis	Chronic	571.42
Skin	Chronic	
Alopecia areata	Chronic	704.01
Dermatitis herpetiformis and other bullous dermatoses	Chronic	694[c]
Discoid lupus	Chronic	695.4
Localized scleroderma and other hypertrophic and atrophic conditions of skin	Chronic	701.0, 701.2, 701.3
Psoriasis and similar disorders	Chronic	696 [c]
Gastrointestinal	Chronic	
Celiac disease	Chronic	579.0
Regional enteritis/Crohn's disease	Chronic	555 [c]
Pernicious anemia	Chronic	281.0

© 2022 Schonfeld SJ et al. *JAMA Network Open.*

Ulcerative colitis	Chronic	556[c]
Hematologic	Chronic	
Autoimmune hemolytic anemia	Chronic	283.0
Immune thrombocytopenic purpura	Chronic	287.31
Nervous system	Chronic	
Multiple sclerosis	Chronic	340
Guillain-Barre syndrome	Chronic	357.0
Myasthenia gravis	Chronic	358.0
Miscellaneous		
Asthma	Chronic	493[c]
Autoimmune disease, not otherwise specified	Chronic	279.4
II. Other immune-related AEs	Transient	
Cardiovascular	Transient	
Myocarditis (includes acute, subacute)	Transient	422.0, 422.9, 429.0
Pericarditis	Transient	420[c]
Endocrine	Chronic	
Cushing's syndrome[d]	Chronic	255.0
Thyrotoxicosis with or without goiter (hyperthyroidism)	Chronic	242[c]
Hypopituitarism	Chronic	253.2, 253.7
Hypothyroidism	Chronic	244[c]
Thyroiditis (acute thyroiditis, subacute thyroiditis, iatrogenic thyroiditis, thyroiditis unspecified)	Chronic	245.1, 245.4, 245,9
Other disorders of the pituitary gland (includes hypophysitis)	Chronic	253.8
Eye	Transient	
Ocular myositis	Transient	376.1
Optic neuritis	Transient	377.3
Uveitis and Iritis	Transient	364.0, 360.11
Gastrointestinal		
Gastroenteritis and colitis excluding ulcerative colitis	Transient	009.0, 009.1, 558
Diarrhea	Transient	787.9
Pancreatitis	Transient	577.0, 577.1
Stomatitis and mucositis (includes ulcerative, aphthous)	Transient	528.0, 528.2
Hepatitis (chronic hepatitis unspecified, chronic persistent hepatitis)	Chronic	573.3, 571.40
Myalgia and myositis, not otherwise specified	Transient	729.1

© 2022 Schonfeld SJ et al. *JAMA Network Open.*

Pneumonitis	Transient	516.32, 516.33
Vitiligo	Chronic	709.01
Septicemia, sepsis, septic shock[e]	Transient	785.52, 995.9

a. For each outcome, we identified the first claim [Claim types: MEDPAR, Carrier claims (NCH), OUTPT, and DME)] at or before 1st primary melanoma diagnosis as well as the first claim after first primary melanoma diagnosis.
b. Immune-related AEs were designated as "chronic" or "transient". For chronic diseases, it is assumed that an individual can only have one incident diagnosis per lifetime. For transient diseases, it is assumed that an individual can have multiple independent events in the lifetime.
c. Includes all codes beyond the decimal point.
d. Although not typically considered among immune-related AEs, we included this as rare events have been reported after immune checkpoint inhibitors (Reference 20 in the main text).
e. Although not typically considered among immune-related AEs, we included this as rare events have been reported after immune checkpoint inhibitors (References 2,3 in the main text).

© 2022 Schonfeld SJ et al. *JAMA Network Open.*

eTable 3. Hazard ratios for selected immune-related adverse events (AEs) after ipilimumab only and after any immune checkpoint inhibitor (ICI) - among patients diagnosed with stages III-IV melanoma, by time since first ICI, and by baseline history of autoimmune disease[a]

	**After ipilimumab – all patients**	**After any ICI – restricted to stages III-IV melanoma**	**By time since first ICI claim – all patients**			**By baseline history of autoimmune disease – all patients**		
			< 6 months	6+ months		Yes	No	
	HR (95% CI)[b]	HR (95% CI)[b]	HR (95% CI)[b,c]	HR (95% CI)[b],[c]	P het [d]	HR (95% CI)[b,e]	HR (95% CI)[b],[e]	P het.[f]
I. Autoimmune-related AEs	2.7 (1.7-4.3)	1.8 (1.0-3.2)	3.2 (2.0-5.2)	1.4 (0.7-2.9)	0.02	N.E	N.E	N.E
Endocrine	9.1 (4.4-18.6)	6.7 (2.9-15.6)	9.2 (4.3-19.7)	8.1 (3.5-19.1)	0.75	8.3 (3.2-21.3)	10.2 (4.7-22.2)	0.34
Primary adrenal insufficiency	10.4 (4.8-22.6)	7.2 (2.8-18.3)	9.8 (4.2-22.7)	10.0 (4.0-24.9)	0.96	8.0 (2.9-21.9)	11.2 (4.8-26.0)	0.43
Gastrointestinal	3.7 (1.7-8.0)	2.7 (1.0-7.1)	5.7 (2.7-12.0)	N.E.	<0.001	5.8 (2.1-16.0)	3.1 (1.3-7.3)	0.42
Ulcerative colitis	8.8 (2.9-26.5)	5.2 (1.3-21.7)	15.8 (5.3-47.1)	N.E.	<0.001	N.E.	11.1 (3.3-38.0)	0.31
II. Other immune-related AEs	2.2 (1.8-2.9)	1.9 (1.4-2.6)	3.0 (2.3-3.8)	0.7 (0.4-1.1)	<0.001	2.0 (1.4-2.8)	2.5 (2.0-3.3)	0.02
Endocrine	3.3 (2.0-5.2)	3.2 (1.8-5.6)	4.9 (3.1-7.7)	1.1 (0.5-2.3)	<0.001	4.0 (2.1-7.7)	3.4 (2.0-5.6)	0.75
Hypopituitarism	20.4 (5.6-74.3)	14.9 (3.4-66.1)	24.8 (6.4-95.6)	12.5 (2.6-61.1)	0.31	22.6 (4.4-116.5)	19.5 (4.8-80.1)	0.87
Hypothyroidism	3.8 (2.4-6.1)	3.8 (2.2-6.8)	5.5 (3.5-8.7)	1.5 (0.8-3.1)	<0.001	4.9 (2.6-9.0)	3.8 (2.3-6.3)	0.84
Gastrointestinal	3.2 (2.3-4.3)	2.5 (1.7-3.7)	5.1 (3.7-7.0)	0.6 (0.3-1.2)	<0.001	2.6 (1.7-4.1)	3.6 (2.6-5.2)	0.02
Diarrhea	3.8 (2.7-5.2)	3.4 (2.2-5.2)	5.9 (4.2-8.3)	0.7 (0.3-1.4)	<0.001	3.4 (2.1-5.5)	4.1 (2.8-5.9)	0.10
Myalgia and myositis, nos	1.1 (0.6-2.3)	1.4 (0.6-3.1)	2.2 (1.1-4.3)	N.E.	0.02	3.4 (1.6-7.1)	0.9 (0.4-2.3)	0.07
Septicemia, sepsis	2.2 (1.4-3.4)	1.9 (1.2-3.1)	3.3 (2.1-5.1)	0.9 (0.5-1.7)	<0.001	1.4 (0.8-2.5)	3.0 (1.9-4.8)	0.004

© 2022 Schonfeld SJ et al. *JAMA Network Open.*

Abbreviations: ICI, immune checkpoint inhibitor; AE, adverse event; HR, hazard ratio; CI, confidence interval; N.E., not estimated; NOS, not otherwise specified

a. Study population restricted to white patients diagnosed with AJCC stage II-IV (or unknown stage) cutaneous melanoma during 2011-2015 as identified in SEER-Medicare linked database. Selected outcomes include those with at least 11 events after ICIs from the full analysis presented in Table 2.
b. Hazard ratios (HRs) and 95% Confidence intervals (CIs) estimated from multivariable Cox Proportional hazards regression with person-years as the time scale and stratified by calendar year of melanoma diagnosis. All models adjusted for age at melanoma diagnosis (66-69, 70-74, 75-79,80-84), sex, stage at melanoma diagnosis, NCI comorbidity index, (0, >0-1 - <1.69, 1.69+; cutpoints derived from individuals with non-zero values), and time-dependent variables for history of autoimmune and non-autoimmune disease; chemotherapy, radiotherapy, and other types of immunotherapy. Models of transient outcomes further adjusted for baseline history of that disease. HRs suppressed if N < 5.
c. HRs by time since first receipt of ICI include the variables described in b and a time-dependent variable for ICI classified as none, <6 months since first claim and 6+ months since first claim.
d. P-value for heterogeneity by time since first ICI based on likelihood ratio test comparing models b and c.
e. HRs by baseline history of autoimmune disease estimated from model described in b with the addition of a main effect for baseline history of autoimmune disease and interaction term between ICI and baseline history of autoimmune disease.
f. P-value for heterogeneity by baseline history of autoimmune disease based on likelihood ratio test comparing model b (with additional parameter for baseline history of autoimmune disease and model e.

© 2022 Schonfeld SJ et al. *JAMA Network Open.*

eTable 4. Cumulative incidence of selected immune-related adverse events (AEs) by time (years) accounting for competing risk of death.

	After ICI[a]			No ICI/Prior to ICI[b]		
**Event**	**Cumulative incidence (95% CI)**			**Cumulative incidence (95% CI)**		
	**6 months**	**1 year**	**2 years**	**6 months**	**1 year**	**2 years**
A. Total study population						
Autoimmune-related AEs	13.7 (9.7-18.3)	18.5 (13.7-23.8)	20.9 (15.6-26.8)	4.5 (3.8-5.3)	7.0 (6.1-8.1)	11.0 (9.7-12.4)
Primary adrenal insufficiency	4.5 (2.8-7.0)	8.0 (5.4-11.3)	10.5 (7.3-14.5)	0.4 (0.2-0.6)	0.6 (0.4-0.9)	1.1 (0.8-1.5)
Ulcerative colitis	3.7 (2.1-6.0)	3.7 (2.1-6.0)	4.3 (2.5-7.0)	0.2 (0.1-0.4)	0.3 (0.2-0.6)	0.6 (0.4-1.0)
Other immune-related AEs	46.8 (40.7-52.7)	52.0 (45.7-57.9)	55.0 (48.3-61.1)	24.3 (23.0-25.6)	32.7 (31.2-34.2)	43.1 (41.4-44.9)
Diarrhea	23.3 (19.1-27.8)	25.2 (20.8-29.9)	27.1 (22.3-32.1)	3.4 (2.9-4.0)	5.9 (5.2-6.7)	10.4 (9.3-11.5)
Hypothyroidism	16.6 (12.3-21.5)	21.0 (16.1-26.3)	23.2 (17.8-29.0)	3.1 (2.5-3.8)	5.2 (4.4-6.1)	9.0 (7.8-10.3)
Hypopituitarism	2.5 (1.2-4.5)	4.0 (2.2-6.5)	4.5 (2.5-7.3)	0.1 (0.1-0.3)	0.2 (0.1-0.4)	0.3 (0.1-0.5)
Septicemia, sepsis	12.1 (9.0-15.6)	14.1 (10.8-17.9)	17.3 (13.3-21.8)	2.8 (2.3-3.3)	4.3 (3.7-5.0)	7.0 (6.1-7.9)
B. Restricted to patients diagnosed with AJCC Stages III-IV melanoma						
Autoimmune-related AEs	11.0 (6.9-16.3)	17.7 (12.1-24.1)	20.9 (14.6-28.0)	5.5 (4.2-7.2)	7.7 (6.0-9.7)	12.4 (10.0-15.2)
Primary adrenal insufficiency	2.8 (1.3-5.3)	7.0 (4.2-10.7)	9.6 (6.0-14.1)	0.5 (0.2-1.1)	0.6 (0.3-1.3)	1.4 (0.7-2.4)
Ulcerative colitis	2.9 (1.4-5.5)	2.9 (1.4-5.5)	3.7 (1.8-6.9)	0.1 (0.0-0.4)	0.3 (0.1-0.8)	0.6 (0.2-1.3)
Other immune-related AEs	46.0 (38.8-52.9)	51.3 (43.8-58.3)	54.4 (46.5-61.5)	27.3 (24.8-29.9)	34.7 (31.9-37.5)	45.5 (42.2-48.8)
Diarrhea	23.7 (18.7-29.0)	25.8 (20.6-31.3)	27.2 (21.7-32.9)	4.5 (3.4-5.8)	6.8 (5.4-8.4)	11.6 (9.5-13.9)
Hypothyroidism	16.6 (11.6-22.5)	20.5 (14.9-26.8)	23.4 (17.2-30.3)	3.9 (2.7-5.3)	6.0 (4.4-7.8)	10.5 (8.1-13.2)
Hypopituitarism	2.6 (1.2-5.1)	4.1 (2.1-7.2)	4.1 (2.1-7.2)	0.4 (0.1-0.9)	0.6 (0.2-1.2)	0.7 (0.3-1.4)
Septicemia, sepsis	12.5 (9.0-16.7)	14.8 (10.8-19.3)	16.9 (12.4-21.9)	5.4 (4.2-6.8)	7.5 (6.0-9.1)	9.8 (8.0-11.9)

Abbreviations: ICI, immune checkpoint inhibitor; adverse events, AEs; CI, confidence interval

a. Cumulative incidence after ICI captures person-time beginning at date of first ICI claim and continues until earliest of the event of interest or end of follow-up as defined in the text.

© 2022 Schonfeld SJ et al. *JAMA Network Open.*

b. Cumulative incidence in the "no ICI/prior to ICI" group captures person-time from date of melanoma diagnosis until first ICI claim (for those who received an ICI), event of interest, or end of follow-up as defined in the text. Cumulative incidence in the absence of ICI is provided for perspective but it should be noted that the curves have different time scales and are not adjusted for differences between the populations and should therefore be interpreted cautiously.

© 2022 Schonfeld SJ et al. *JAMA Network Open.*

eFigure 2. Cumulative incidence of selected autoimmune- and other- immune-related adverse events (AEs) among patients diagnosed with stages III-IV cutaneous melanoma, accounting for competing risk of death, by receipt of immune checkpoint inhibitors (ICIs).[a]

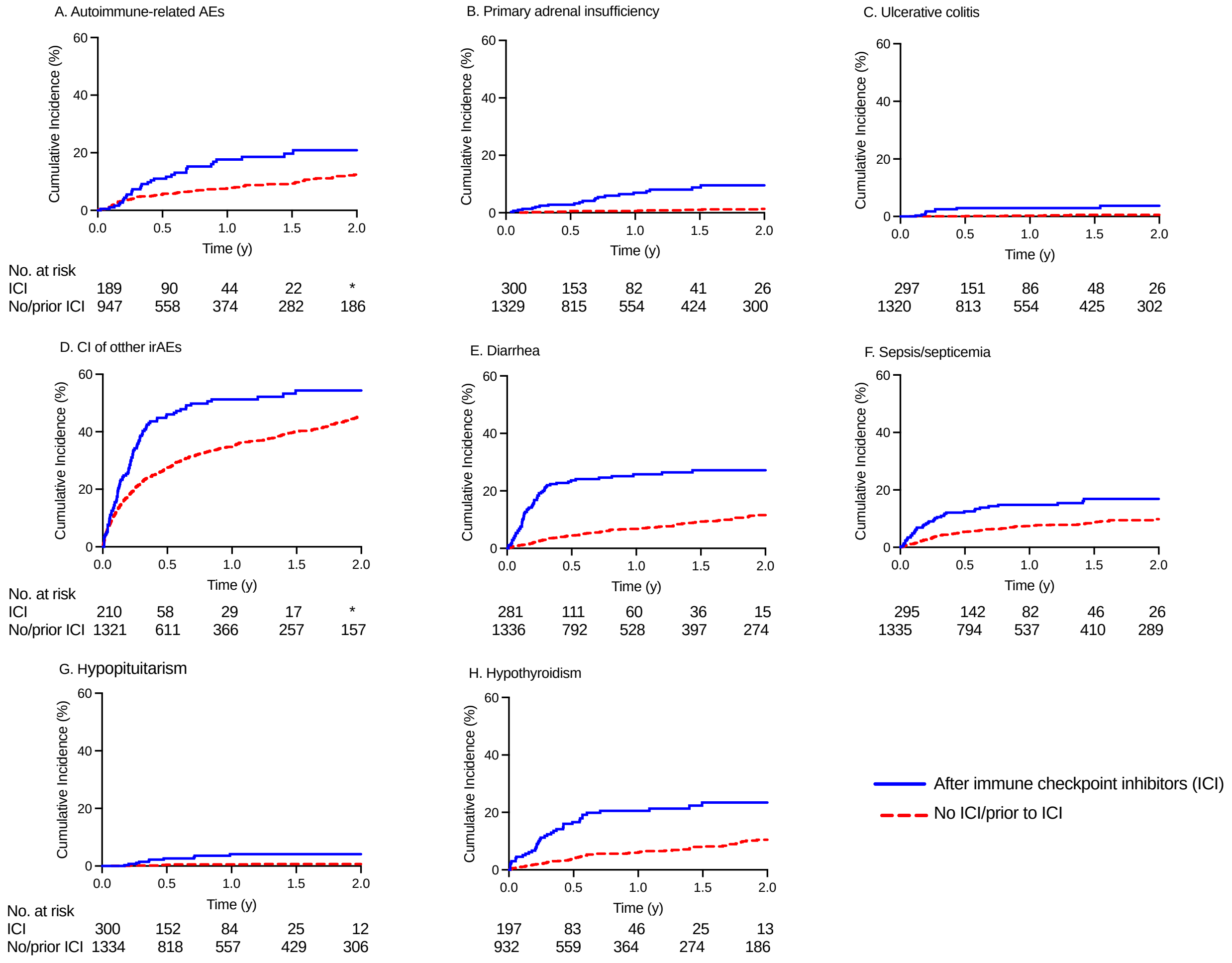

a. See eTable 2 for definition of autoimmune- (panels A-C) and other- (panels D-H) immune-related AEs. Below each figure, “Number at risk” specifies the number of people at-risk at the start of each time interval. Small numbers suppressed for privacy. Cumulative incidence in the absence of immune checkpoint inhibitors (ICI) is provided for perspective but it should be noted that the curves have different time scales and are not adjusted for differences between the populations and should therefore be interpreted cautiously. See Supplement eTable 4 for point estimates and 95% confidence bounds. Individuals with a previous claim (at/prior to melanoma diagnosis) were excluded from analyses of autoimmune immune-related AEs (overall), primary adrenal insufficiency, ulcerative colitis, hypothyroidism, and hypopituitarism, as described in online eTable 2. For all outcomes, the number at risk at the start of an interval excludes people who had the event earlier in follow-up or died or ended follow-up prior to the start of the interval.

© 2022 Schonfeld SJ et al. *JAMA Network Open.*
